# MPT0G612, a Novel HDAC6 Inhibitor, Induces Apoptosis and Suppresses IFN-γ-Induced Programmed Death-Ligand 1 in Human Colorectal Carcinoma Cells

**DOI:** 10.3390/cancers11101617

**Published:** 2019-10-22

**Authors:** Mei-Chuan Chen, Yu-Chen Lin, Yu-Hsuan Liao, Jing-Ping Liou, Chun-Han Chen

**Affiliations:** 1 Ph.D. Program in Clinical Drug Development of Herbal Medicine, College of Pharmacy, Taipei Medical University, Taipei 110, Taiwan; mcchen1250@tmu.edu.tw (M.-C.C.); a0916143813@gmail.com (Y.-H.L.); 2 Traditional Herbal Medicine Research Center of Taipei Medical University Hospital, Taipei 110, Taiwan; 3 Department of Pharmacology, School of Medicine, College of Medicine, Taipei Medical University, Taipei 110, Taiwan; amylin0083@tmu.edu.tw; 4 School of Pharmacy, College of Pharmacy, Taipei Medical University, Taipei 110, Taiwan; jpl@tmu.edu.tw; 5 Cell Physiology and Molecular Image Research Center, Wan Fang Hospital, Taipei Medical University, Taipei 110, Taiwan

**Keywords:** HDAC6, apoptosis, autophagy, PD-L1, colorectal cancer

## Abstract

Colorectal cancer (CRC) is the third most common cancer and the leading cause of cancer-associated death worldwide. Histone deacetylases (HDACs) have been implicated in regulating complex cellular mechanisms to influence tumor biology and immunogenicity in various types of cancer. The potential of selective inhibition of HDAC6 has been widely discussed for the treatment of hematologic malignancies. We previously identified that MPT0G612 is a novel HDAC6 inhibitor exhibiting a promising antitumor activity against several solid tumors. The purpose of the present study was to evaluate the feasibility and pharmacological mechanisms of MPT0G612 as a potential therapy for CRC patients. Results revealed that MPT0G612 significantly suppresses the proliferation and viability, as well as induces apoptosis in CRC cells. Autophagy activation with LC3B-II formation and p62 degradation was observed, and the inhibition of autophagy by pharmacological inhibitor or Atg5 knockdown enhances MPT0G612-induced cell death. In addition, HDAC6 knockdown reduces MPT0G612-mediated autophagy and further potentiates apoptotic cell death. Furthermore, MPT0G612 downregulates the expression of PD-L1 induced by IFN-γ in CRC cells. These results suggest that MPT0G612 is a potent cell death inducer through inhibiting HDAC6-associated pathway, and a potential agent for combination strategy with immune checkpoint inhibitors for the treatment of CRC.

## 1. Introduction

Epigenetic gene regulation with histone modifications confers alteration of cell phenotypes and intracellular signaling [1,2]. Targeting epigenetic regulations in cancer has become one of the promising strategies to improve current treatments [3]. Histone deacetylases (HDACs) are one of the major epigenetic enzymes that function as acetyl group removers of lysine residues from histones or non-histone protein substrates [4]. Since elevated expression level and activity of HDACs have been observed in numerous cancer types, inhibition of HDAC activity appears to be a promising approach for anticancer therapy [5]. The first generation of HDAC inhibitors, such as Vorinostat and Romidepsin, have been approved for cutaneous T cell lymphoma (CTCL) therapy [6]. However, these agents have shown limited activity in solid tumors, partially due to their unpleasant toxicities and difficulties for combining them with other agents [7].

There are 18 HDAC enzymes divided into four major classes in humans: class I (HDACs 1, 2, 3, 8), class IIa (HDACs 4, 5, 7, 9), class IIb (HDACs 6 and 10), and class IV (HDAC 11) are Zn^2+^-dependent metalloenzymes, while class III Sir2-like proteins (SIRTs 1–7) are NDA^+^-dependent sirtuins [8]. It is notable that targeting ubiquitously expressed HDAC1 and HDAC2 may lead to severe toxicities [9]. Embryonic lethality, severe proliferation defects, and general growth retardation can be observed in HDAC1-null mice [9,10]. HDAC2-null mice will develop severe cardiac malformations and will die within the first 24 h after birth [9]. In addition, previous reports have shown non-selective HDAC inhibitors lead to several mild to severe side effects [11,12]. Therefore, development of new HDAC inhibitors for preclinical evaluation will be needed to focus on improving HDAC isoform selectivity and efficacy against solid tumors. It is believed that selective targeting on HDAC6 may have fewer side effects than inhibition of pan-HDAC and class I isoforms [13,14]. Current HDAC 6 inhibitors include Tubastatin A and ACY-1215 that have been tested as a preclinical investigated compound and for a lot of clinical trials in combination with other drugs, respectively [15,16,17].

Colorectal cancer (CRC) is the third most common cancer and the leading cause of cancer-associated death worldwide [18]. Although the treatment of CRC with 5-fluorouracil (5-FU)-based chemotherapy has been used for standard therapy for decades, development of resistance to 5-FU, high recurrence, and low survival rate remain challenging tasks to overcome [19]. Several pieces of evidences have shown intervention of epigenetic modification may benefit therapeutic outcomes [20,21,22]. Combination of HDAC6 inhibitor ACY-1215 with oxaliplatin or 5-FU has shown to increase the efficacy of conventional chemotherapy, suggesting HDAC 6 inhibitor may play a role in improving therapeutic strategy for CRC treatment [20,23].

Our previous study has revealed a novel HDAC6 inhibitor, 3-[4-(3-dimethylaminomethyl-2-methyl-indole-1-sulfonyl)-phenyl]-N-hydroxy-acrylamide (compound 9, hereafter named MPT0G612), which has better selectivity and potency than ACY-1215 and tubastatin A, exhibiting a promising antitumor activity against several solid tumors [24]. In the current study, we aim to examine the anticancer effect and the pharmacological mechanisms of MPT0G612 in CRC cells.

## 2. Results

### 2.1. MPT0G612 Suppresses Cell Proliferation and Viability in Colorectal Cancer Cells

**Figure 1:**
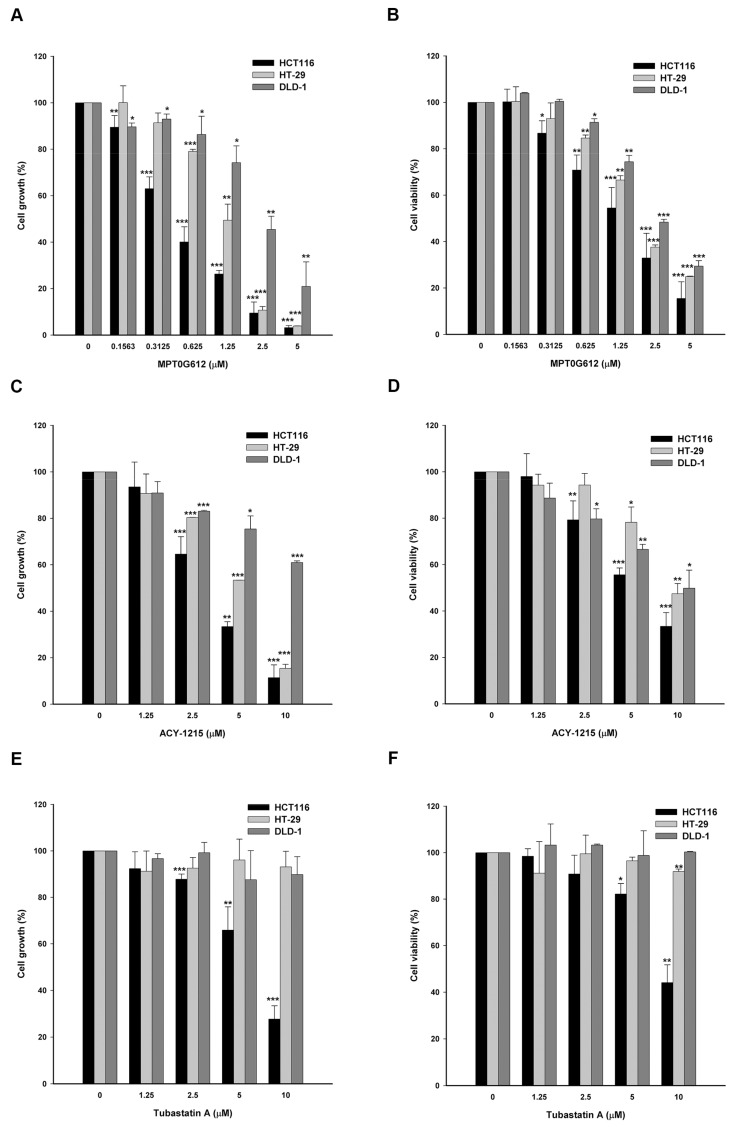
MPT0G612 inhibits cell proliferation and cell viability in CRC cells. MPT0G612 suppresses cell proliferative activity (**A**) and cell viability (**B**) in a concentration-dependent manner in HCT116, HT-29, and DLD-1 cells. The inhibitory effects of ACY-1215 and Tubastatin A on cell proliferation (**C**,**E**) and viability (**D**,**F**) in HCT116, HT-29, and DLD-1 cells. Cells were treated with or without the indicated concentrations of drugs for 48 h, and cell growth and cell viability were evaluated by SRB and MTT assay. Data are expressed as means ± S.D. of at least three independent experiments. * *p* < 0.05; ** *p* < 0.01; and *** *p* < 0.001 compared with the control group.

We have previously reported that ring-opened tetrahydro-γ-carbolines display selectivity to HDAC6 with promising inhibitory effects on several solid tumors [24]. In this study, we focus on the mechanism of action and exerting abilities of MPT0G612 in CRC cells. By performing SRB and MTT assays, the data revealed that MPT0G612 exhibits significant activity on inhibition of proliferation and viability in HCT116 cells with GI_50_ and IC_50_ values of 0.5 ± 0.1 µM and 1.6 ± 0.54 µM, respectively (Figure 1A,B). We also included reference compounds ACY-1215 and Tubastatin A to compare with MPT0G612. As shown in Figure 1C,E, the GI_50_ of ACY-1215 and Tubastatin A is 3.6 ± 0.3 µM and 6.8 ± 1.1 µM respectively. Meanwhile, the IC_50_ for ACY-1215 and Tubastatin A is 6.3 ± 0.56 µM and 9.3 ± 0.94 µM, respectively (Figure 1D,F). Similar results were observed that MPT0G612 displays higher potency than ACY-1215 and Tubastatin A in HT-29 and DLD-1 cells (Figure 1A–F; Supplementary Table S1). These results indicate that MPT0G612 shows higher potency than ACY-1215 and Tubastatin A in inhibiting cell growth and viability in CRC cells.

### 2.2. MPT0G612 Induces Cell Cycle Accumulation at subG1 Phase and Apoptosis in CRC Cells

**Figure 2:**
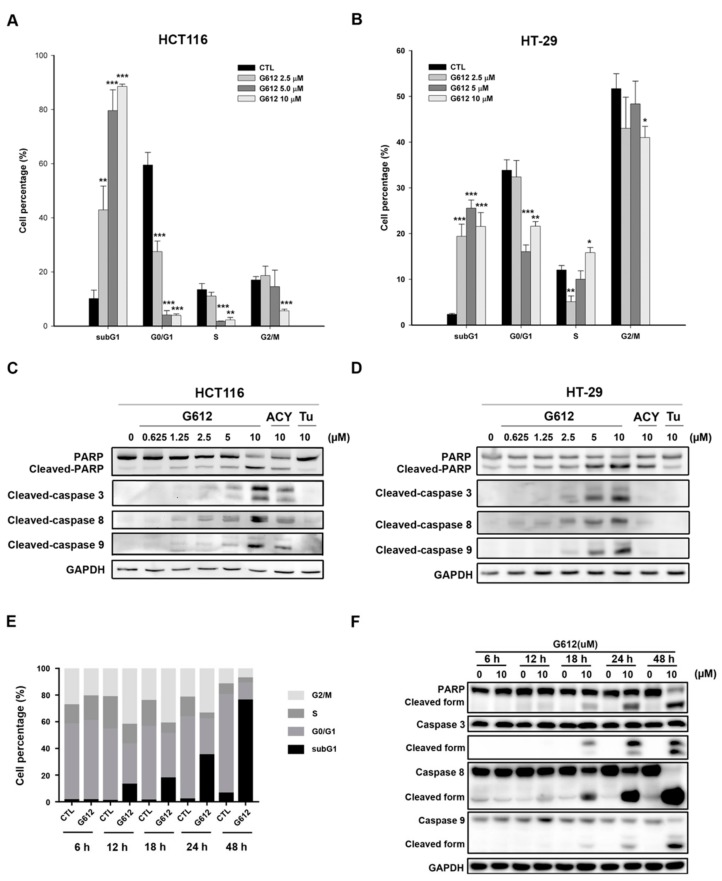
MPT0G612 induces subG1 cell accumulation and apoptosis in CRC cells. MPT0G612 enhances accumulation of subG1 phase in HCT116 (**A**) and HT-29 (**B**) cells. Cells were treated with indicated concentrations of MPT0G612 (G612) for 48 h, then the cell cycle distribution was analyzed by flow cytometry. Quantitative data are based on flow cytometry histograms, and presented as means ± S.D. of at least three independent experiments. * *p* < 0.05; ** *p* < 0.01; and *** *p* < 0.001 compared with the control (CTL) group. MPT0G612 (G612) induces more significant apoptosis than ACY-1215 (ACY) and Tubastatin A (Tu) in CRC cells. HCT-116 (**C**) and HT-29 (**D**) cells were treated with the indicated concentrations of compounds for 48 h, and cell lysates were immunoblotted using the indicated antibodies. (**E**,**F**) MPT0G612 induces subG1 cell accumulation and apoptosis in a time-dependent manner. (E) HCT116 cells were treated with MPT0G612 (G612; 10 μM) for indicated times, then the cell cycle distribution was analyzed by flow cytometry. Quantitative data are based on flow cytometry histograms, and presented as means of at least three independent experiments. (F) MPT0G612 time-dependently induced apoptotic cell death. HCT116 cells were treated with MPT0G612 (G612; 10 μM) for indicated times, and subjected to immunoblotted using the indicated antibodies.

Next, we performed propidium iodide (PI) staining to examine the alteration of the cell cycle progression by drug treatments. Previous study indicates HDAC 6 inhibitor ACY-1215 shows induction of cell death mainly occurring after 48 h treatment in solid tumors [25]. Indeed, the pronounced elevation of subG1 cell population was detected in response to 48 h treatment of MPT0G612 in HCT116 and HT-29 cells (Figure 2A,B). MPT0G612-induced apoptosis was further confirmed by increased population of Annexin V^+^/PI^+^ in HCT116 and HT-29 cells (Supplementary Figure S1). However, MPT0G612 activated more significant apoptotic cell death-related proteins (cleaved PARP and caspase 3/8/9) than ACY-1215 and Tubastatin A did after 48 h treatment (Figure 2C,D). We further performed time-course experiments to measure the effect of MPT0G612 on apoptosis in HCT116 cells. The results showed that MPT0G612 increases significant subG1 phase accumulation after 18–48 h treatment in HCT116 cells (Figure 2E). The time points were parallel to apoptosis induction as evidenced by the activation of caspase3/8/9 and PARP (Figure 2F). Taken together, MPT0G612 exhibits a strong capacity to trigger cell death at an earlier time point which provides better therapeutic efficacy than usual HDAC6 inhibitors do in CRC cells.

### 2.3. Effects of MPT0G612 on Autophagy in HCT116 Cells

**Figure 3:**
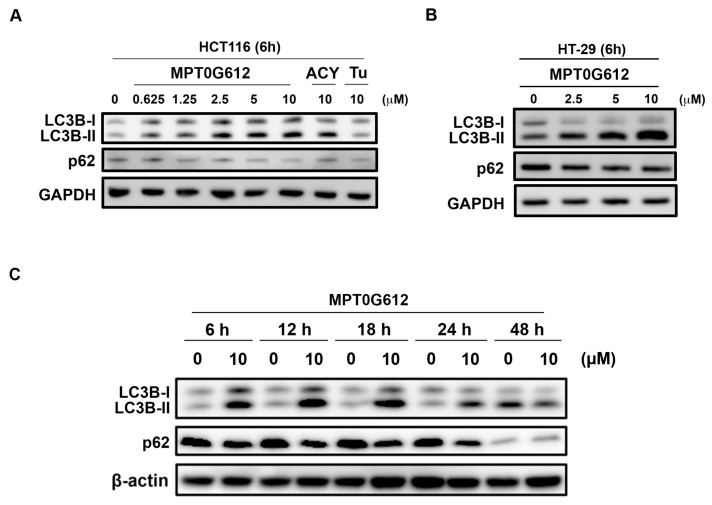
MPT0G612 induces autophagy in a concentration-and time-dependent manner in CRC cells. HCT116 (**A**) and HT-29 (**B**) cells were exposed to indicated concentrations of MPT0G612, ACY-1215 (ACY) or Tubastatin (Tu) for 6 h. The cell lysates were subjected to western blot analysis. (**C**) The cells were treated with MPT0G612 (10 μM) for indicated times, and subjected to western blot analysis.

It has been reported that HDAC6 inhibition may context-dependently interfere with autophagy pathways or autophagic flux [22,26]. We further examine two critical autophagic biomarkers LC3B-II and p62 to evaluate autophagy in response to drug treatment. As shown in Figure 3A, 6 h treatment of MPT0G612 and ACY-1215 triggers LC3B-II formation and downregulation of p62, suggesting these compounds activate autophagy in HCT116 cells. On the other hand, tubastatin A did not show an obvious change on the expression of LC3-II and p62 in HCT116 cells (Figure 3A), suggesting MPT0G612 and ACY-1215 may have a similar effect on the regulation of autophagy. MPT0G612 also concentration-dependently increases LC3B-II formation and decreases p62 levels in HT-29 cells (Figure 3B). In order to better understand the dynamic change of autophagy after drug treatment, the expression levels of autophagic proteins were determined in response to MPT0G612 treatment for different time points. We found an early autophagy event was activated at 6 h of MPT0G612 treatment, and the activation of autophagic biomarkers (LC3-II and p62) persisted to 24 h, suggesting 6–24 h is the critical period for MPT0G612 to activate autophagy in HCT116 cells (Figure 3C).

### 2.4. Inhibition of Autophagy Enhances MPT0G612-Induced Cell Death

**Figure 4:**
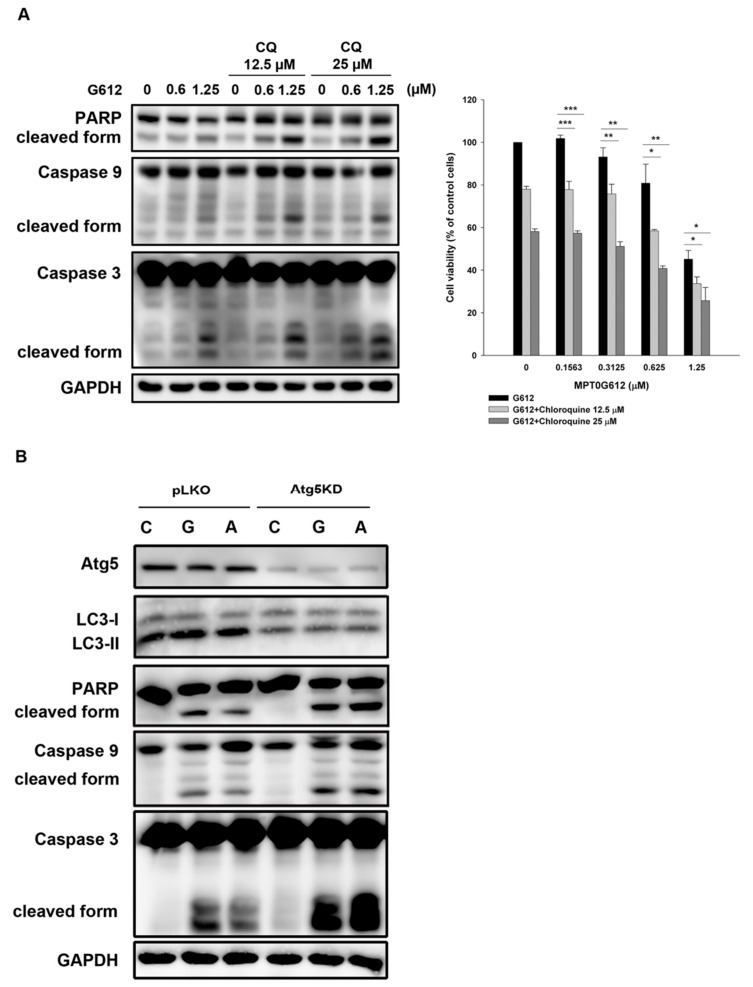
MPT0G612 induces pro-survival autophagy in HCT116 cells. (**A**) Blockade of autophagy augments MPT0G612-induced apoptosis. The cells were treated with MPT0G612 (G612) in the presence or absence of chloroquine (CQ) for 48 h, and protein lysates were analyzed by western blot analysis with indicated antibodies (left panel). Cell viability was determined by MTT assay, and the data were presented as means ± S.D. of at least three independent experiments (right panel). * *p* < 0.05; ** *p* < 0.01; and *** *p* < 0.001 compared with MPT0G612 treatment alone. (**B**) Knockdown of Atg5 potentiated MPT0G612-induced apoptosis. The cells were transduced with control vector (pLKO) or shRNA against Atg5 (Atg5KD) by lentivirus, and stable cells were obtained by puromycin selection (2 μg/mL). The cells were then treated with vehicle (C), MPT0G612 (G, 10 μM) or ACY-1215 (A, 10 μM) for 48 h, and subjected to western blot analysis by using indicated antibodies.

To determine the role of autophagy in MPT0G612-induced cytotoxicity, we combined MPT0G612 with pharmacological inhibitor of autophagy or genetically knockdown (KD) of autophagy regulator Atg5 to observe whether the cell survival will be impaired by autophagy inhibition. Co-treatment with chloroquine (CQ) enhances MPT0G612-induced apoptotic cell death, as evidenced by activated caspase 3/9 and PARP (Figure 4A, left panel). Cell viability was further suppressed by the combination of CQ with MPT0G612 (Figure 4A, right panel). Besides, Atg5 KD resulted in less LC3-II formation and more significant cell death induction than control vector (pLKO) in MPT0G612 and ACY-1215-treated cells (Figure 4B), suggesting autophagy is likely an essential factor for survival in HCT116 cells. These findings show blockade of autophagy potentiates MPT0G612-induced apoptotic cell death in HCT116 cells, suggesting autophagy acts as a pro-survival signal to control downstream regulations.

### 2.5. HDAC6 Is Crucial to MPT0G612-Induced Apoptosis and Autophagy in CRC Cells

**Figure 5:**
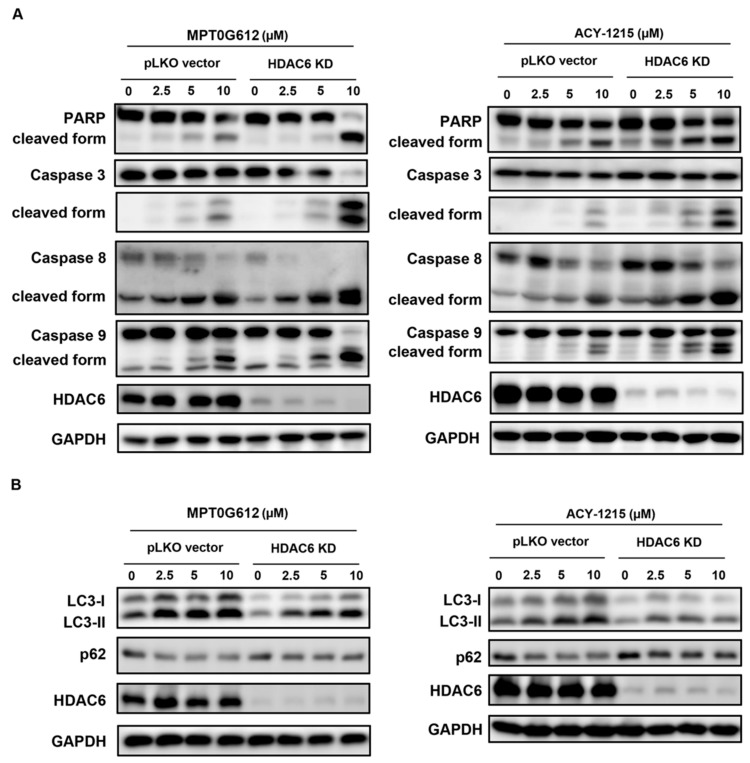
HDAC6 plays a regulatory role in induction of apoptosis and autophagy. (**A**) Knockdown of HDAC6 augments apoptosis induced by MPT0G612 or ACY-1215 in HCT116 cells. The cells were transduced with control vector (pLKO) or shRNA against HDAC6 (HDAC6 KD) by lentivirus, and stable cells were obtained by puromycin selection (2 μg/mL). The cells were exposed to indicated concentrations of MPT0G612 (left panel) or ACY-1215 (right panel) for 48 h and subjected to western blotting. (**B**) Knockdown of HDAC6 rescues autophagy induced by MPT0G612 or ACY-1215 in HCT 116 cells. The cells stalely express control vector (pLKO) or shRNA against HDAC6 were treated with indicated concentrations of MPT0G612 (left panel) or ACY-1215 (right panel) for 48 h and subjected to western blotting.

To evaluate whether MPT0G612-mediated cytotoxicity is an on-target effect, we genetically knocked down HDAC6 and examined the effects on MPT0G612 treatment in HCT116 cells. As shown in Figure 5A, HDAC6 knockdown sensitized MPT0G612-and ACY-1215-induced apoptosis, as evidenced by dramatical increase of cleaved caspase 3/8/9 and PARP. These results demonstrated a chemical-genetic interaction [27], suggesting HDAC6 is the target of MPT0G612-and ACY-1215-induced apoptosis. Previous reports have indicated HDAC6 may context-dependently activate or inhibit autophagy pathways [26,28]. In order to understand the association between HDAC6 and autophagy in response to drug treatment, we utilized HDAC6 KD cells to investigate the role of HDAC6 in MPT0G612-mediated autophagy. As shown in Figure 5B, MPT0G612-and ACY-1215-induced LC3B-II formation and p62 suppression were reversed in HDAC6 KD cells, indicating HDAC6 is required for autophagy activation. Taken together, knockdown of HDAC6 prevents autophagy and further potentiates cell death in MPT0G612-treated HCT116 cells.

### 2.6. MPT0G612 Abrogates IFN-γ-Induced PD-L1 Expression in CRC Cells

**Figure 6:**
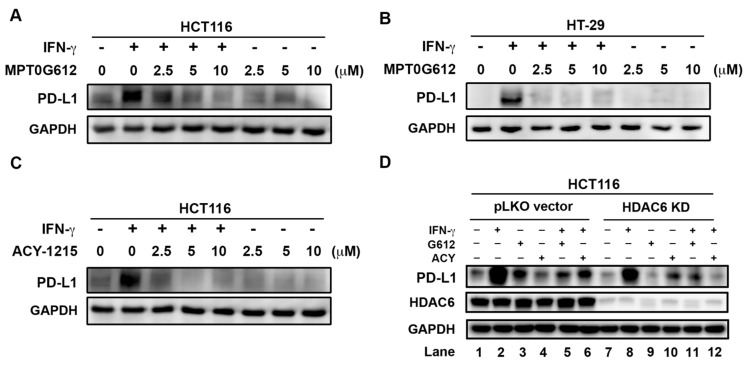
MPT0G612 inhibits IFN-γ-induced PD-L1 expression in CRC cells. MPT0G612 (**A**,**B**) and ACY-1215 (**C**) abrogated IFN-γ-induced PD-L1 levels in HCT116 or HT-29 cells. The cells were treated with indicated concentrations of compounds in the presence or absence of IFN-γ (10 ng/mL) for 48 h, and subjected to western blot analysis. (**D**) HCT116 cells stalely express control vector (pLKO) or shRNA against HDAC6 were treated with MPT0G612 (G612) or ACY-1215 (ACY) in the presence or absence of IFN-γ (10 ng/mL) for 48 h, and protein levels of PD-L1 was examined by western blotting.

Previous reports have shown inhibition of HDAC6 leads to decrease immunosuppression and enhance T-cell immune properties in melanoma patient cells [29]. Meanwhile, HDAC6 is essential in the regulation of PD-L1 expression in melanoma cells [30]. In order to investigate whether MPT0G612 affects the expression level of PD-L1, we measured the effect of MPT0G612 on IFN-γ-induced PD-L1 expression in CRC cells by western blot analysis. The data showed that PD-L1 level induced by IFN-γ (10 ng/mL) was abrogated by MPT0G612 in a concentration-dependent manner in HCT116 and HT-29 cells (Figure 6A,B). A similar result was observed when HCT116 cells were treated with ACY-1215 (Figure 6C). Interestingly, both of MPT0G612 and ACY-1215 could inhibit IFN-γ-induced PD-L1 expression level in pLKO control vector and HDAC6 KD HCT116 cells (Figure 6D, lane 5, 6, 11, and 12). We also found IFN-γ induced less PD-L1 production in HDAC6 KD cells than in control pLKO vector cells (Figure 6D, lane 8 vs. lane 2). Lower expression of PD-L1 can be detected across the entire HDAC6 KD group than the pLKO control vector group (Figure 6D, lane 7–12 vs. lane 1–6). These results indicate HDAC6 may contribute to inducible PD-L1 expression and also be a potential target for downregulation of PD-L1 to further benefit the outcome of immune checkpoint inhibitors.

## 3. Discussion

Targeting HDACs as a promising strategy to deal with anticancer treatment has been studied for decades [31,32]. Although several pan-HDAC inhibitors (e.g., vorinostat, romidepsin, and panobinostat) are currently in clinical use, those drugs were mainly approved for cutaneous T-cell lymphoma (CTCL) or multiple myeloma therapy instead of solid tumors [33,34,35]. Also, previous reports have shown numerous cytotoxicity due to the lack of selectivity of pan-HDAC inhibitors [36,37]. Panobinostat (LBH589) displays highly potent anticancer against a broad spectrum of HDACs, accelerating its approval for multiple myeloma in combination with bortezomib and dexamethasone [38]. However, considerable numbers of patients had difficulties to tolerate this combination therapy and led to treatment discontinued because of undesired side effects during clinical trials [39]. These results indicate the balance between efficacy and toxicity becomes one of the most important challenges in drug development.

Specific targeting on HDAC6 soon receives a lot of attention and has been applied to numerous diseases treatments [40,41,42]. HDAC6 is reported to interact with microtubule-associated protein tau and is likely to be responsible for acetylation–phosphorylation switch of tau to affect neurological diseases progression [43,44]. Neuritic beading is considered to be an early hallmark of neuronal toxicity and correlates with degenerating synapses in Alzheimer’s disease (AD) [45]. Inhibition of HDAC6 suppresses neuritic tau bead formation in vivo, suggesting targeting on HDAC6 may bring some benefits to stop the onset of neurological disorders [46]. On the other hand, HDAC6 functions as a critical player to participate in the process of tumorigenesis through oncogenic cell transformation and tumor cell migration and invasion [47,48].

ACY-1215 is classified as a selective HDAC6 inhibitor and also show potent anticancer activity mainly in hematologic malignancies [17]. Several reports also demonstrated ACY-1215 exhibits promising anticancer activity in numerous solid tumors [25,49]. However, some studies have reported that ACY-1215 is not potent enough as monotherapy for some solid tumors, such as the treatment of colorectal cancer (CRC), ovarian cancer, and breast cancers which generally requires combination therapy for successful treatment [20,50,51]. Our study represents a novel HDAC6 inhibitor to show better potency and potential than ACY-1215 to become a single agent for CRC therapy. We identified MPT0G612 as a potent cell death inducer to generate more accumulation amounts of activated caspase 3/8/9 and PARP than ACY-1215 in CRC HCT116 cells. In addition, MPT0G612 induces LC3-II expression level and p62 downregulation to activate autophagy pathway. Inhibition of autophagy by the combination of chloroquine (CQ) or Atg5 KD potentiates MPT0G612-induced cytotoxicity, indicating the role of autophagy induction may play a pro-survival role in cells. Of note, chloroquine could potentiate MPT0G612-induced apoptosis under lower concentrations (Figure 4A). Therefore, it warrants a rational combination of MPT0G612 with chloroquine for the therapy of CRC in the future. Previous studies have demonstrated autophagy may context-dependently affect cell fate decision, indicating a different underlying mechanism of autophagy induction may lead to contradictory results [52,53]. Knockdown of HDAC6 attenuates autophagy activation by showing decreased LC3B-II and increased p62 in MPT0G612 and ACY-1215-treated cells, suggesting HDAC6 has a regulatory role in autophagy activation. In addition, HDAC6 KD can further potentiate MPT0G612-induced cell death. These results suggest HDAC6 may be a pivotal mechanism for autophagy and cell death in CRC. On the other hands, the unique characteristics of HDAC6 included management of protein aggregates by having an intrinsic ubiquitin-binding activity and associates with both microtubules and the actin cytoskeleton [54,55,56,57]. In addition, the association of HDAC6 and actin regulates autophagosome-lysosome fusion to affect the degradative capacity of autophagy in cells [26]. These distinct cellular properties of HDAC6 from other HDAC isoforms make it a potential therapeutic target for diseases associated with misfolded proteins.

There is a growing trend to use checkpoint inhibitor-based immunotherapies to make immune cells recognize and attack tumor cells [58,59]. Revolutionized care has been widely discussed to combine different cancer treatments since checkpoint inhibitors do not present clinical effectiveness all the time [60,61]. It has been reported that HDAC inhibition can increase program death ligand-1 (PD-L1) and augments the efficacy of PD-1 blockade in melanoma cancer [62]. In addition, several studies identified HDAC inhibition to be a promising therapeutic strategy to potentiate immunotherapy in tumor cells [63,64]. Loss of HDAC6 activity can also change the gene expression of critical immune system modulators, including PD-1 and PD-L1, which are currently central targets in cancer immunotherapies [65]. Notably, non-selective HDAC inhibitors are known to upregulate PD-L1 on the cell surface of tumor cells while selective HDAC6 inhibitor would show the opposite effect by decreasing the expression of PD-L1 [66,67,68]. Our results showed both of MPT0G612 and ACY-1215 reduced IFN-γ-enhanced PD-L1 in HCT116 cells. Of note, HDAC6 KD slightly reduced IFN-γ-induced PD-L1 compared with control pLKO vector cells. These results suggest HDAC6 plays a role in the expression of PD-L1 in cancer cells. It has been reported that glycosylation of PD-L1 inhibits 26S proteasome-mediated protein degradation to further stabilize PD-L1 protein [69]. Also, the epidermal growth factor receptor (EGFR) inhibitor gefitinib reduces PD-L1 expression level and sensitizes PD-1 blockade therapy [69]. However, the other study showed gefitinib induces PD-L1 expression in resistant non-small-cell lung cancer (NSCLC) cells, resulting in the suppression of T cell function and immune escape [70]. Combination strategies with ERK1/2 pathway inhibitors and PD-L1/PD-1 inhibitors are considered to suppress PD-L1 expression in tumor cells to restore EGFR-TKI sensitivity in resistant cells [70]. These studies suggest a combination of MPT0G612 and immunotherapy may result in a potential clinical outcome and be worth further investigation. Collectively, these results demonstrate MPT0G612 is not only a potential compound for CRC treatment but also for combination strategies in tumors with PD-L1 upregulation.

## 4. Materials and Methods

### 4.1. Cell Lines and Reagents

HCT116 and HT-29 cells were obtained from the American Type Culture Collection (ATCC) (Manassas, VA, USA). DLD-1 cell was a kind gift from Dr. Er-Chieh Cho (School of Pharmacy, College of Pharmacy, Taipei Medical University, Taiwan). Cells were maintained in 10% fetal bovine serum (FBS)-supplemented RPMI 1640 medium and 1% penicillin–streptomycin (GIBCO, Grand Island, NY, USA) at 37 °C in a humidified incubator containing 5% CO_2_. Compound MPT0G612 (3-[4-(3-dimethylaminomethyl-2-methyl-indole-1-sulfonyl)-phenyl]-N-hydroxy-acrylamide) was obtained from Professor Jing-Ping Liou (School of Pharmacy, College of Pharmacy, Taipei Medical University, Taiwan). Puromycin, ACY-1215, tubastatin A, rapamycin, and wortamannin were from Cayman Chemical (Ann Arbor, MI, USA). Anti-mouse and anti-rabbit IgGs were from Jackson Immuno Research Laboratories (West Grove, PA, USA).

### 4.2. Cell Viability Assay

The cells were seeded in 96-well plates and exposed to indicated treatments for 48 h to perform cell viability (MTT) assay that was assayed by the 3-(4,5-dimethylthiazol-2-yl)-2,5-diphenyltetrazolium bromide. Growth inhibition was expressed as the percentage of surviving cells in drug-treated versus DMSO-treated control cells (which was considered as 100% viability). The IC_50_ value was the concentration resulting in 50% cell growth suppression by a 48 h exposure to drug(s) compared with untreated control cells.

### 4.3. SRB (Sulforhodamine B) Assay

Cells were seeded into 96-well plates and cultured overnight followed by the exposure to various concentrations of indicated drugs for 48 h. Cells were then fixed in situ with 10% trichloroacetic acid (TCA) to represent a measurement of the cell population at the time of drug addition (T_0_). After an additional 48 h incubation with or without tested compound in medium with 5% FBS, the assay was terminated by 10% TCA. SRB dye pursed from Sigma (St. Louis, MO, USA) at 0.4% (w/v) in 1% acetic acid was added to stain the cells. Unbound dye was removed by 1% acetic acid and the plates were air dried. Bound dye was subsequently solubilized with 10 mM trizma base, and the absorbance was read at a wavelength of 515 nm. Cell growth percentage and GI_50_ values were calculated as described previously [24].

### 4.4. FACScan Flow Cytometric Analysis

Cells were seeded in 6-well plates (2.5 × 10^5^/well) and treated with DMSO or indicated compounds with various concentrations for indicated times. Cells were washed with phosphate-buffered saline, fixed in ice-cold 70% ethanol at −20 °C overnight, and stained with propidium iodide (80 μg/mL) containing Triton X-100 (0.1%, v/v) and RNase A (100 μg/mL) in phosphate-buffered saline. DNA content was analyzed with the FACScan and CellQuest software (Becton Dickinson, Mountain View, CA, USA). For detection of apoptosis, the cells were harvested by trypsinization and stained with Annexin V-FITC/PI (Thermo Fisher Scientific, Waltham, MA, USA) as previously described [71].

### 4.5. Western Blot and Lentivirus Expression System

Cells were seeded in dishes and allowed to attach for overnight. The cells were treated with drugs at indicated concentrations for indicated times. After the indicated exposure time, cells were lyzed and the immunoblotting was performed as previous described [72]. The whole blots were shown in Supplementary Data. Antibodies against various proteins were obtained from the following sources: PARP (poly-ADP-ribose polymerase) and p62, were obtained from Santa Cruz (Dallas, TX, USA). Caspase 8, capspase 9, and Atg5 were obtained from Cell Signaling Technology (Danvers, MA, USA). Beta-actin was purchased from Millipore (Billerica, MA, USA). Caspase 3 and LC3B were purchased from Novus (Littleton, CO, USA). Lentiviral particles containing plasmids of shRNA against Atg5 (TRCN0000151474, TRCN0000151963) and HDAC6 (TRCN0000004842, TRCN0000314910) were purchased from the National RNAi Core Facility (Academia Sinica, Taipei, Taiwan). The cells were seeded in a 6-well plate, and transduced with two clones of lentiviruses for each gene. Stable cells were obtained by the selection of puromycin (2 μg/mL).

### 4.6. Statistics and Data Analysis

Each experiment was performed at least three times, and presentative data are shown. Data in bar graph are given as the means ± S.D. Means were checked for statistical difference using the *t*-test and *p*-values less than 0.05 were considered significant (* *p* < 0.05, ** *p* < 0.01, *** *p* < 0.001).

## 5. Conclusions

In conclusion, our study reveals that MPT0G612 exhibits higher potency than other selective HDAC6 inhibitors (ACY-1215, tubastatin A) in inhibiting of cell growth and triggering apoptosis. Meanwhile, we observed that MPT0G612 is able to block IFN-γ-induced PD-L1 expression in CRC cells. Together, we provide the pharmacological mechanisms of MPT0G612, fostering new therapeutic strategies for the therapy of CRC as a single agent or in combination with immune checkpoint inhibitors in the future.

## Supplementary Materials

The following are available online at https://www.mdpi.com/2072-6694/11/10/1617/s1, Figure S1: Effects of MPT0G612 on apoptosis in CRC cells. Table S1: The GI_50_ and IC_50_ values of different drugs in HCT116, HT-29 and DLD-1 cells.
