# Supplementary material for: MPT0G612, a Novel HDAC6 Inhibitor, Induces Apoptosis and Suppresses IFN-γ-Induced Programmed Death-Ligand 1 in Human Colorectal Carcinoma Cells
Source: Cancers (Basel). 2019 Oct 22;11(10):1617. doi: 10.3390/cancers11101617 (PMC6826904; doi:10.3390/cancers11101617)
Supplement: Supplementary file 1 [file cancers-11-01617-s001.pdf]

Supplementary Materials:

MPT0G612, a Novel HDAC6 Inhibitor, Induces Apoptosis and Suppresses IFN-γ-Induced Programmed Death-Ligand 1 in Human Colorectal Carcinoma Cells

Mei-Chuan Chen, Yu-Chen Lin, Yu-Hsuan Liao, Jing-Ping Liou and Chun-Han Chen

Table S1. The GI_{50} and IC_{50} values of different drugs in HCT116, HT-29 and DLD-1 cells.

Compound	HCT116		HT-29		DLD-1	
	GI_{50} (μM)	IC_{50} (μM)	GI_{50} (μM)	IC_{50} (μM)	GI_{50} (μM)	IC_{50} (μM)
MPT0G612	0.5 ± 0.1	1.6 ± 0.54	1.27 ± 0.16	1.97 ± 0.01	2.30 ± 0.25	2.43 ± 0.06
ACY-1215	3.6 ± 0.3	6.3 ± 0.56	5.45±0.02	9.56±0.75	> 10	>10
Tubastatin A	6.8 ± 1.1	9.3 ± 0.94	> 10	>10	> 10	>10

Fig. S1

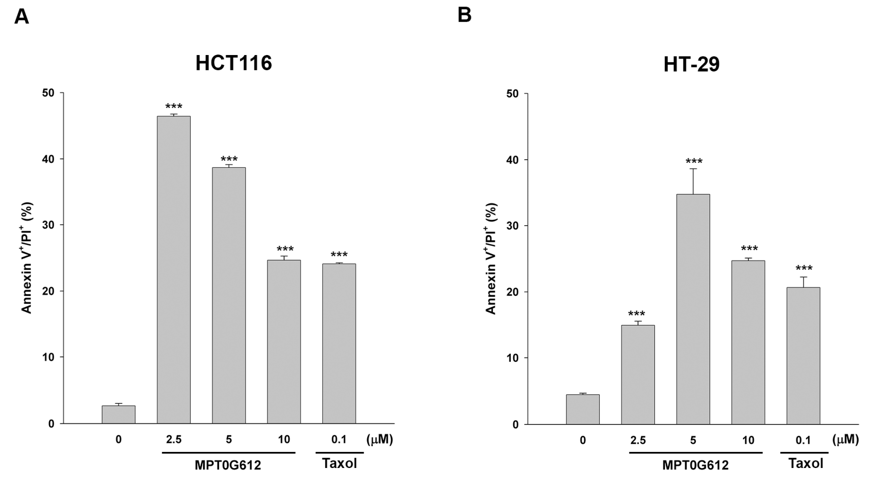

Figure S1. Effects of MPT0G612 on apoptosis in CRC cells. HCT116 (**A**) and HT-29 (**B**) cells were exposed to indicated concentrations of MPT0G612 for 48h. The cells were then stained with Annexin V-FITC/PI solution and analyzed by flow cytometry. Paclitaxel (Taxol, 0.1 μM) was included as a positive control of apoptosis. *** $p < 0.001$.

Western blots

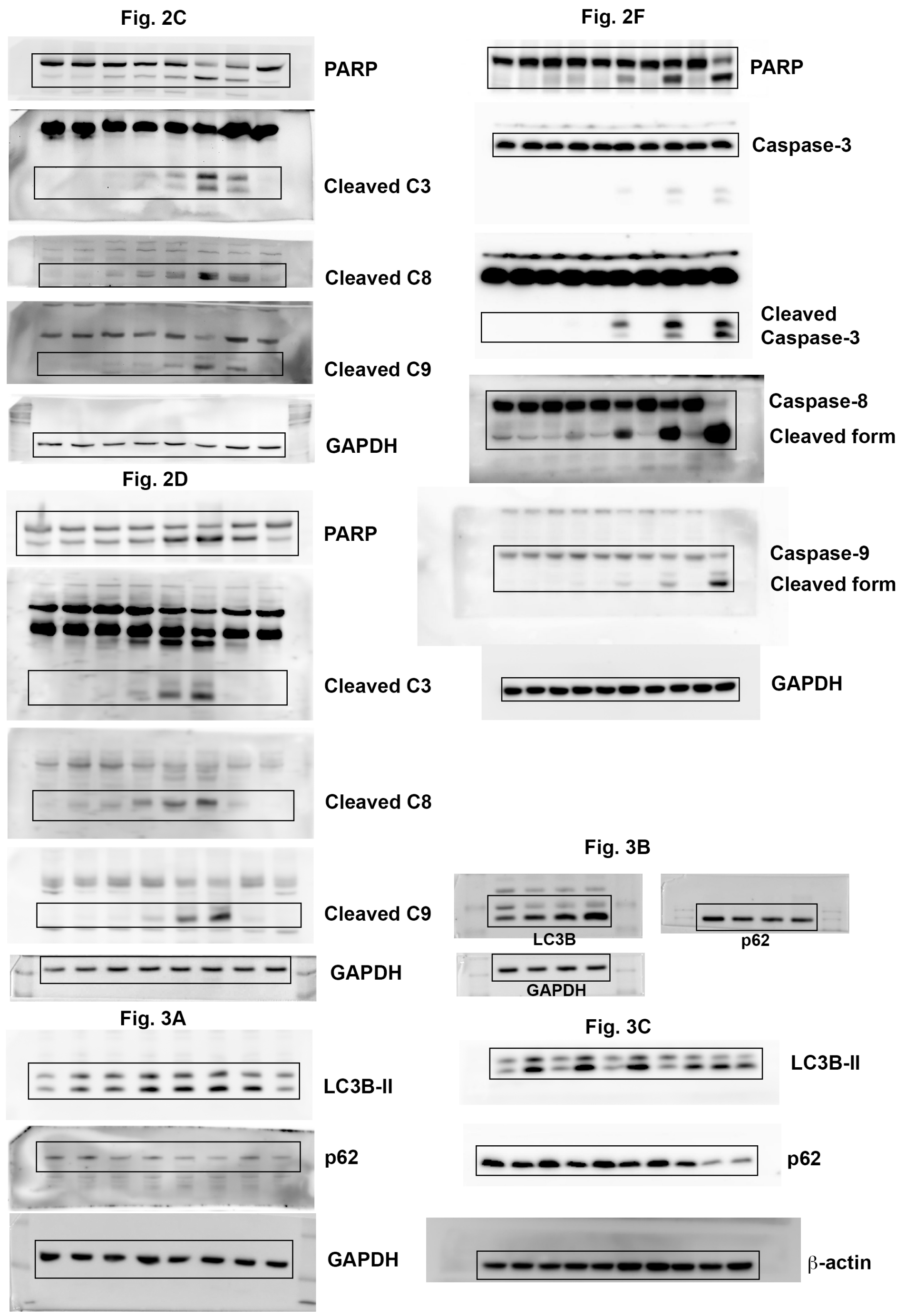

Fig. 4A

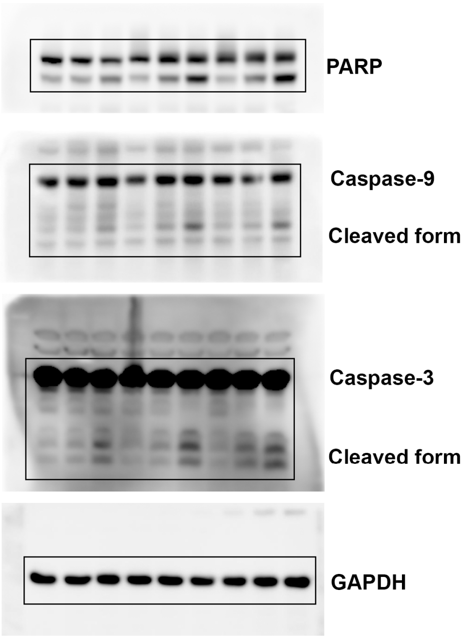

Fig. 4B

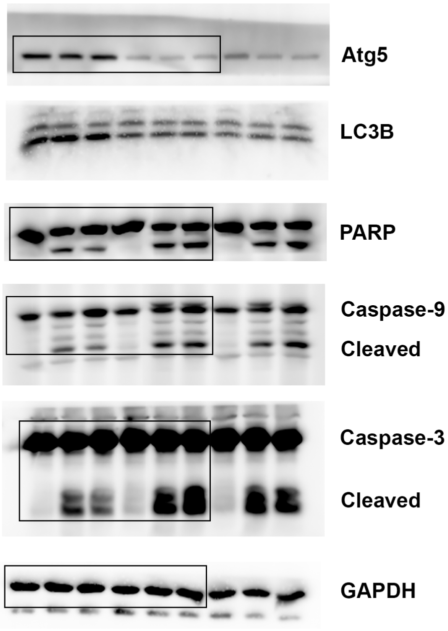

Fig. 5A (left panel)

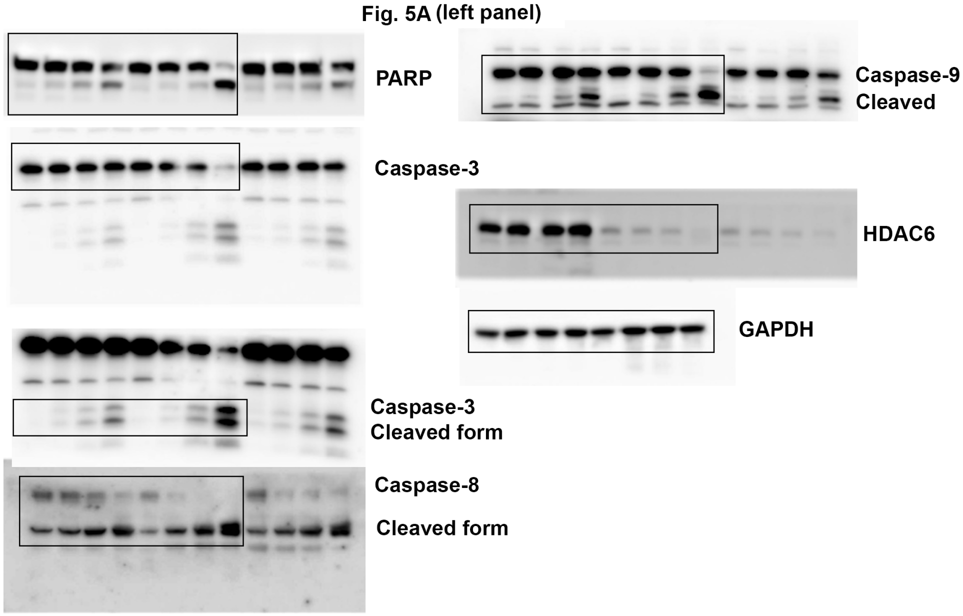

Fig. 5A (right panel)

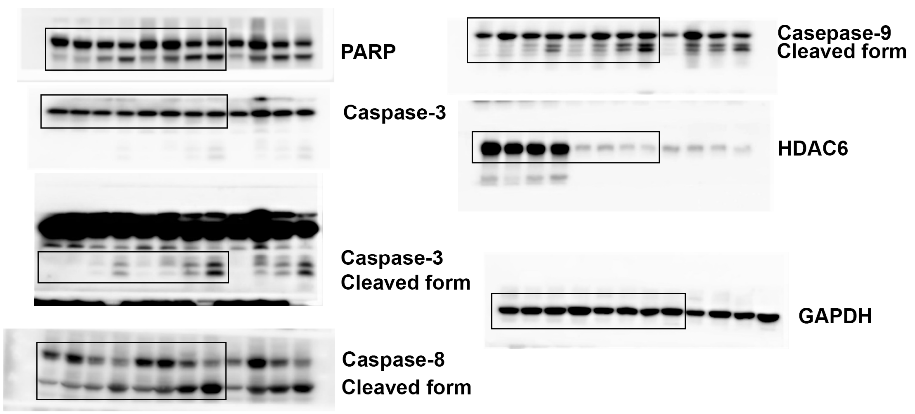

Fig. 5B (left panel)

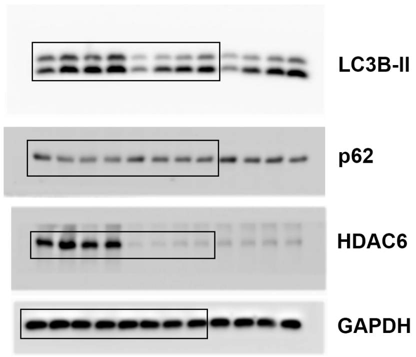

Fig. 5B (right panel)

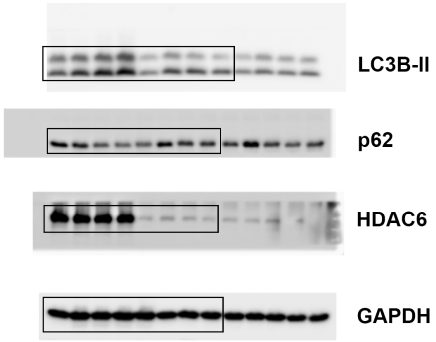

Fig. 6A

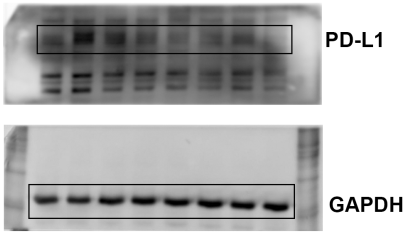

Fig. 6B

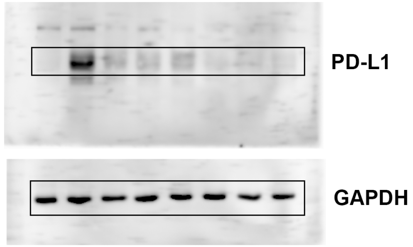

Fig. 6C

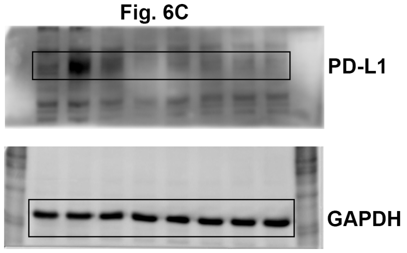

Fig. 6D

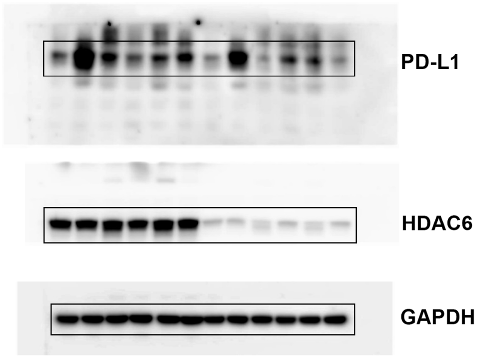

© 2019 by the authors. Licensee MDPI, Basel, Switzerland. This article is an open access article distributed under the terms and conditions of the Creative Commons Attribution (CC BY) license (http://creativecommons.org/licenses/by/4.0/).
